# ASSOCIATIONS BETWEEN MULTIPLE SOURCES OF SUPPORT AND STRAIN AND SELF-PERCEPTIONS OF AGING

**DOI:** 10.1093/geroni/igae098.1527

**Published:** 2024-12-31

**Authors:** Gina Lee, William Chopik

**Affiliations:** University of Wisconsin Madison, Madison, Wisconsin, United States; Michigan State University East Lansing, East Lansing, Michigan, United States

## Abstract

How people perceive the aging process is associated with important health, well-being, and cognition outcomes. For example, people with more positive self-perceptions of aging (SPA) live longer and have better cognition than those with negative self-perceptions (Levy et al., 2002; Tully-Wilson et al., 2021). One underappreciated source of variation in these aging perceptions might arise from our immediate social context—namely, the close relationships we find ourselves in. However, previous research has tested this in a piecemeal fashion, often neglecting the multiple relationships that we inhabit at once. The current study examined changes and variations in SPA and how these perceptions vary according to sources of support and strain from spouses, children, family, and friends. We used random intercept cross-lagged panel models to model within-person changes in SPA and support/strain over twelve years (2008-2020) among 22,160 older adults from the Health and Retirement Study. The results indicated that strain is more closely tied to SPA than support. Spousal support and SPA had positive reciprocal associations. SPA negatively predicted spousal strain, but not vice versa. Interestingly, higher strain from other family members and friends shaped more positive SPA, whereas support from these sources was largely unrelated to SPA. Child support/strain was not associated with SPA. Such findings provide a better understanding of how different close relationships shape aging perceptions. Future studies can test different mechanisms and outcomes underlying each relational contributor of SPA by exploring cognitive, psychological, and/or physiological factors.

